# Knowledge and Practice of the Preventive and Care Methods for Diabetic Foot Among the Caregivers of Diabetic Patients in Saudi Arabia

**DOI:** 10.7759/cureus.37887

**Published:** 2023-04-20

**Authors:** Hassan Ali A AlZubaidi, Ali Nori O Alfaqih, Mohammed Hussain A Alothayqi, Hassan Mohammed H Alfaqih, Alaa Jameel A Albarakati, Medhat Taha, Abdulkarim M Alnashri

**Affiliations:** 1 Department of Medicine and Surgery, Umm AlQura University, Al-Qunfudah College of Medicine, Al-Qunfudhah, SAU; 2 Department of Surgery, Umm AlQura University, Al-Qunfudah College of Medicine, Al-Qunfudhah, SAU; 3 Department of Anatomy, Umm AlQura University, Al-Qunfudah College of Medicine, Al-Qunfudhah, SAU; 4 Department of Anatomy and Embryology, Mansoura University, Mansoura, EGY

**Keywords:** saudi arabia, diabetic patients, caregiver, diabetes, diabetic foot

## Abstract

Background

Diabetic foot syndrome is a complex and multifactorial disease process involving neuropathy, peripheral arterial disease, osteomyelitis, diabetic foot ulcer (DFU), and amputation. DFUs are a common and burdensome manifestation of the syndrome, responsible for diabetes-related morbidity and mortality. Successful management of DFU requires collaboration between patients and caregivers. This study assesses the knowledge, experience, and practices of the caregivers of diabetic foot patients in Saudi Arabia, highlighting the need for targeted interventions to improve knowledge and practices in certain subgroups of caregivers.

Method

The primary objective of this study was to evaluate the proficiency and practicality of caregivers who provide care to patients with diabetic foot in the Kingdom of Saudi Arabia. To accomplish this, a cross-sectional study was conducted among caregivers of diabetic foot patients who were aged 18 years or older and living in Saudi Arabia. The participants were randomly chosen to ensure that the sample was representative. The data collection process involved the distribution of a structured online questionnaire via various social media platforms. Prior to the distribution of the questionnaire, the participants were informed about the study's objectives, and their informed consent was obtained. Additionally, adequate measures were taken to ensure the confidentiality of the participants and their caregiving status.

Results

Among the initial pool of 2990 participants, 1023 individuals were excluded from the study due to their status as non-caregivers of diabetic patients or being under the age of 18 years. Consequently, the final sample size consisted of 1921 caregivers. The majority of the participants were female (61.6%), married (58.6%), and had a bachelor's degree (52.4%). The findings revealed that 34.6% of caregivers were attending to patients with diabetic foot, of which 8.5% reported poor foot status and 9.1% reported amputation. Caregivers reported examining the patient's feet in 75.2% of cases, and the feet were cleaned and moisturized by either the patient or caregiver. Nails were trimmed by 77.8% of caregivers, and 49.8% of them did not permit patients to walk barefoot. Moreover, knowledge of diabetic foot care was positively correlated with being female, having a post-graduate degree, having personal experience with diabetes, caring for a patient with diabetic foot, and having prior experience in treating diabetic foot. Conversely, lower knowledge levels were associated with divorced or unemployed caregivers and those residing in the northern region.

Conclusion

The present study highlights that caregivers of diabetic foot patients in Saudi Arabia possess a satisfactory level of knowledge and follow appropriate practices regarding foot care. Nonetheless, it is imperative to identify specific subgroups of caregivers who may require additional education and training to improve their knowledge and practices concerning diabetic foot care. The results of this study could potentially inform the design of tailored interventions aimed at reducing the significant burden of morbidity and mortality attributed to diabetic foot syndrome in the Saudi Arabian context.

## Introduction

Diabetes is a chronic metabolic disorder characterized by hyperglycemia, which causes various pathologies, including microvascular and macrovascular complications, as classified by the International Diabetes Federation (IDF) [1]. Diabetes has now emerged as a major global healthcare emergency, affecting approximately 10.5% of the world population in 2021, with an expected rise to 783.2 million by 2045, including 239.7 million undiagnosed cases [1]. In Saudi Arabia, the prevalence of diabetes was estimated at 13.4% of the total population in 2014 (n=1,745,532), with 959 thousand prediabetic cases [2]. Diabetes is responsible for around 12.2% (6.7 million) of global mortality, and the estimated cost of diabetes in 2021 was approximately $966 billion, which is expected to rise to $1.03 trillion by 2030 and $1.05 trillion by 2045, according to IDF estimations [1].

One of the most serious complications of diabetes is diabetic foot syndrome, which is responsible for most of the morbidity and mortality associated with diabetes [3]. Diabetic foot syndrome is a complex series of pathological processes involving neuropathy, peripheral arterial disease, osteomyelitis, diabetic foot ulcer (DFU), and eventually amputation [3]. DFUs are one of the most common manifestations of diabetic foot syndrome, causing a significant burden to patients, caregivers, and health systems alike [4-6]. DFUs are defined as full-thickness wounds situated below the ankle, regardless of their duration, associated with neuropathy and/or peripheral arterial disease of the lower limb in patients with diabetes. DFUs frequently arise due to multiple factors, mainly neuropathy, along with peripheral arterial disease or trauma [3-7].

It has been estimated that every 30 seconds, a limb is lost due to diabetes, with a diabetic foot incidence rate of 25% in diabetic cases and a global prevalence of approximately 3-8%, rising to 5-7.5% in patients with neuropathy [8,9]. The incidence rate of DFUs in Saudi Arabia is approximately estimated to be 1.8%, with a mean prevalence of 8.5% [10]. Previous research conducted in Jeddah, Saudi Arabia, found that DFUs were responsible for approximately 52.7% of all lower limb amputation (LLA) cases [11].

Several preventive measures have been shown to reduce the incidence of the diabetic foot and the necessity for LLA, including primary health care assessment, patient education on proper self-examination, foot hygiene, and proper footwear, and, most importantly, instructing caregivers on how to evaluate new skin lesions, choose proper footwear, maintain hygiene, and know when to seek medical intervention [3,12,13]. Previous research conducted in Egypt to assess the level of awareness of caregivers about proper DFU care found a variation in the level of knowledge, ranging from insufficient in 56.2% to poor in 46.9% [14]. Another study conducted in Egypt to assess the improvement in the level of foot care practices after the implementation of a foot care enhancement program showed that the majority of caregivers had poor knowledge, but after the program, their knowledge improved substantially [15].

Therefore, this study aims to contribute to the existing literature by assessing the knowledge and practices of caregivers of diabetic patients with DFUs in Saudi Arabia. Understanding the knowledge and practices of caregivers regarding diabetic foot care can help guide the development of targeted interventions to reduce the morbidity and mortality associated with diabetic foot syndrome in Saudi Arabia.

## Materials and methods

This cross-sectional study aimed to evaluate the level of knowledge and foot care practices among caregivers of diabetic patients in Saudi Arabia. The study was conducted over a period of three weeks, from November 11, 2022, to December 1, 2022, with participants selected based on their age (18 years and above), residence in Saudi Arabia, and provision of care to diabetic patients through random sampling.

Questionnaire and data collection

The questionnaire was based on the Diabetic Foot Care Questionnaire of Nova Scotia 2009, as well as prior studies assessing caregivers' knowledge regarding foot care in Egypt [14-16]. It included 30 questions, prefaced by an assurance of participants' confidentiality. The first section comprised two questions seeking consent and differentiating between caregivers and non-caregivers. The second section consisted of seven questions about socio-demographic data, while the final section evaluated the relationship between caregiver and patient, current foot history, knowledge, and foot care practice, including 21 questions. The questions were formed in English and translated into Arabic before distribution. In order to validate the questionnaire and ensure the comprehension of the questions asked, a pilot study was conducted on 100 participants prior to the study's initiation.

The standardized questionnaire was distributed through social media platforms mainly WhatsApp (Whatsapp LLC, Menlo Park, California, United States), Telegram (Telegram Group Inc., Dubai, United Arab Emirates), and Twitter (Twitter, Inc., San Francisco, California, United States) by medical students who were carefully selected to ensure adherence to the required criteria.

Statistical Analysis

Data analysis was performed using RStudio R version 4.1.1 (Released 2021; The R Foundation for Statistical Computing, Vienna, Austria). Categorical variables were presented using frequencies and percentages, while numerical variables were expressed as mean ± standard deviation (SD). Pearson's Chi-squared test was used to assess factors associated with caregivers' knowledge. The significantly associated factors were subsequently entered into a multivariate binary logistic regression analysis to assess the independent predictors of knowledge, with outcomes expressed as odds ratios (ORs) and 95%confidence intervals (CIs). Statistical significance was considered at p < 0.05.

Ethical considerations 

The present study adhered to strict ethical standards and obtained approval from the Research and Ethics Committee of Umm Al-Quran University, Makkah, Saudi Arabia (approval number: HAPO-02-K-012-2022-11-1239). The participants were provided with explicit guarantees of confidentiality during the administration of the questionnaire, in accordance with the principles of research ethics.

## Results

Sociodemographic characteristics

The responses of 2990 participants were initially received on the online system. However, we excluded 1023 responses of caregivers who did not provide care to diabetic patients and 46 responses of those who were aged < 18 years. Therefore, data of 1921 caregivers were included in the current study. The mean ± SD age of participants was 35.5 ± 12.4 years, and participants aged 18-30 years represented 39.9% of the sample. More than half of the respondents were females (61.6%), married (58.6%), and had obtained a Bachelor's degree (52.4%). The majority of participants were Saudis (94.3%). Parents represented the most frequent caregiver (40.1%) (Table 1).

**Table 1:** Sociodemographic characteristics. All values are presented as numbers (N) and percentages (%).

Parameter	Category	N	%
Age (years)	18 to <30	767	39.9%
	30 to <45	639	33.3%
	45 to <60	453	23.6%
	60 or more	62	3.2%
Gender	Male	738	38.4%
	Female	1,183	61.6%
Nationality	Saudi	1,811	94.3%
	Non-Saudi	110	5.7%
Marital status	Single	661	34.4%
	Married	1,126	58.6%
	Divorced	87	4.5%
	Widow	47	2.4%
Region	Eastern	432	22.5%
	Western	268	14.0%
	Northern	336	17.5%
	Southern	330	17.2%
	Central	555	28.9%
Employment status	Student	433	22.5%
	Unemployed	500	26.0%
	Employed	988	51.4%
Educational level	Primary	50	2.6%
	Middle	64	3.3%
	Secondary	397	20.7%
	Diploma	230	12.0%
	Bachelor	1,007	52.4%
	Post-graduate	173	9.0%
Relationship to diabetic patients	Parent	771	40.1%
	Sibling	167	8.7%
	Child	286	14.9%
	Aunt or uncle	200	10.4%
	Grandmother / Grandfather	265	13.8%
	Others	184	9.6%
Do you have a personal history of diabetes	No	1873	97.5%
	Yes	48	2,5%

Characteristics of patients who were treated by the participants

**Figure 1:**
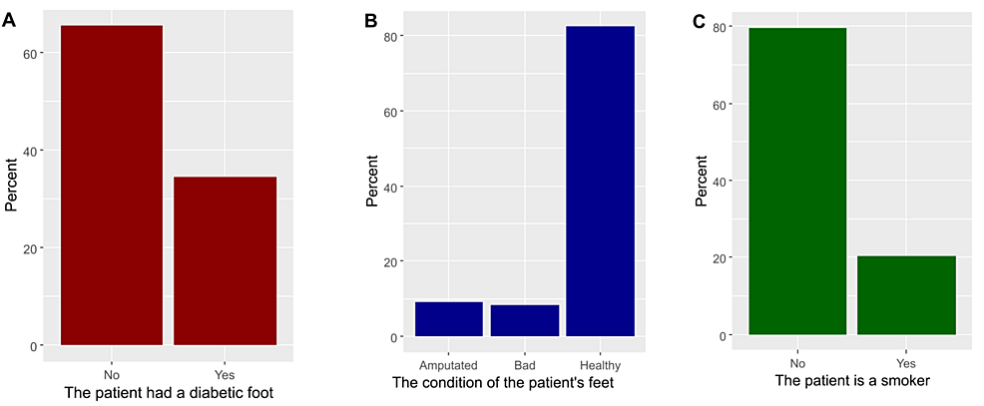
Characteristics of patients who were treated by the participants, including patients who had a diabetic foot (A), status of patients’ feet (B), and smoking status of patients (C).

In general, 34.6% of respondents declared that their patients had diabetic foot (Figure 1A). The status of patients’ feet was bad among 8.5% and amputated among 9.1% (Figure 1B). Almost one-fifth of patients (20.4%) were smokers (Figure 1C). 

Characteristics of patients’ feet care

Generally, the majority of respondents indicated that they checked the patients’ feet (75.2%). The frequency of check-ups was twice or more per week among 21.0% and once weekly among 14.1% of the participants. Furthermore, almost half of the respondents (44.4%) indicated that patients’ feet were always cleaned by the patient or the caregiver. Patients’ feet were also kept moisturized by the patient (51.7%) or the caregiver (25.5%). Focusing on the latter group, the frequency of moisturizing the feet was daily (33.2%) or twice weekly or more (31.1%). Caregivers who had the responsibility of drying patients’ feet after moisturization represented 20.7% of the sample (Table 2). Regarding nail trimming, the majority of participants (77.8%) declared that patients got their nails trimmed and 37.6% of caregivers trimmed the patients’ nails. Approximately half of the respondents indicated that patients were not walking barefoot (49.8%), and they had to ensure that patients wore their shoes (63.7%). The most common types of shoes were the medical (50.2%) and sandal types (31.1%). More than half of the caregivers admitted that patients wore socks with shoes (52.4%), among which cotton socks were the most common (81.6%) (Table 2).

**Table 2:** Characteristics of patients’ feet care. All values are presented as numbers (N) and percentages (%).

Parameter	Category	N	%
Do you check the patient's feet	No	477	24.8%
	Sometimes	550	28.6%
	Yes, once monthly	220	11.5%
	Yes, once weekly	270	14.1%
	Yes, twice or more weekly	404	21.0%
The patient feet are being cleaned, either by the patient or the caregiver	No	328	17.1%
	Rarely	198	10.3%
	Sometimes	542	28.2%
	Always	853	44.4%
The patients’ feet are being moisturized, either by the patient or the caregiver	No	438	22.8%
	Yes, by the patient	992	51.7%
	Yes, by the caregiver	489	25.5%
If you answered yes, how many times do you moisturize the feet	Sometimes	38	7.8%
	Once monthly	30	6.2%
	Once weekly	105	21.6%
	Twice or more weekly	151	31.1%
	Daily	161	33.2%
Are patient's feet dried after getting wet either by the patient or caregiver	No	527	27.5%
	Yes, by the patient	995	51.8%
	Yes, by the caregiver	397	20.7%
Do the patient’s nails get trimmed	No	143	7.4%
	Sometimes	283	14.7%
	Yes	1,495	77.8%
Who trims the patient's nails	The patient	1,144	62.4%
	The caregiver	688	37.6%
Is the patient is walking barefoot	No	957	49.8%
	Sometimes	571	29.7%
	Yes	393	20.5%
Do you make sure that the patient wears his shoes	No	305	15.9%
	Sometimes	393	20.5%
	Yes	1,223	63.7%
What kind of shoes does the patient wear	Medical	964	50.2%
	Sandal	598	31.1%
	Custom	254	13.2%
	Sport	32	1.7%
Does the patient wear socks with shoes	No	447	23.3%
	Sometimes	467	24.3%
	Yes	1,007	52.4%
If yes, what kind of socks	Cotton	797	81.6%
	Wool	137	14.0%
	Silk	43	4.4%

Experience and knowledge regarding diabetic foot

**Figure 2:**
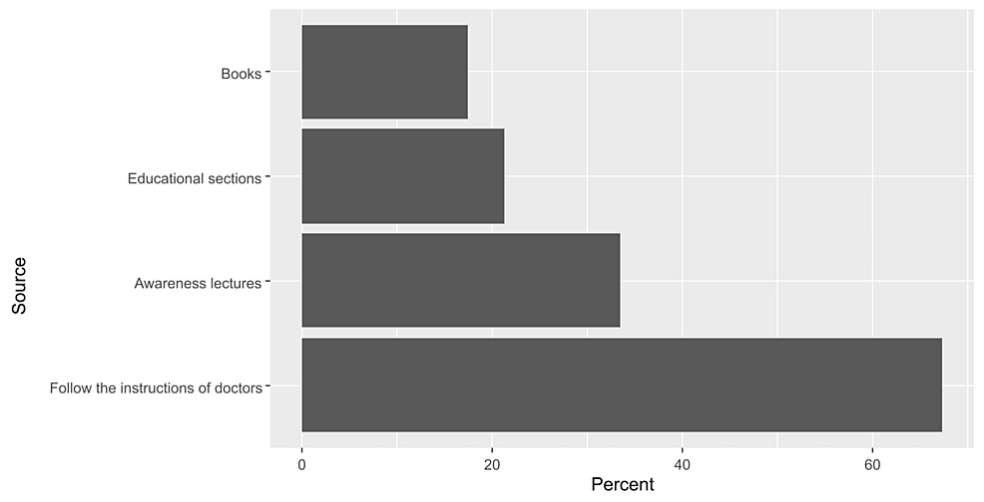
The distribution of sources of information about diabetic foot care.

A total of 936 caregivers (48.7%) confirmed that they had experience in dealing with diabetic patients who had a diabetic foot, among whom the most common sources included instructions from doctors (67.3%) and awareness lectures (33.4%) (Figure 2). Additionally, 1248 caregivers (65.0%) knew about foot complications in diabetic patients (infections/wounds/ulcers/etc.). Knowledge levels differed significantly based on caregivers’ gender (p=0.016), marital status (p<0.001), region of residence (p=0.003), employment status (p<0.001), educational level (p<0.001), relation to the patient (p = 0.025), caring for a patient diagnosed with a diabetic foot (p<0.001), the condition of feet at the time of the study (p<0.001), and having an experience in dealing with a diabetic foot (p<0.001) (Table 3). Based on the regression analysis, knowledge about foot complications in diabetic patients was independently associated with the female gender (OR = 1.42, 95%CI 1.12-1.81, p = 0.004), having a post-graduate degree (OR = 4.92, 95%CI 2.15-11.3, p = 0.004), having a personal history of diabetes (OR = 2.18, 95%CI 1.05-4.82, p = 0.044), caring for a patient diagnosed with a diabetic foot (OR = 2.37, 95%CI 1.78-3.17, p < 0.001) and having an experience in dealing with diabetic foot (OR = 5.28, 95%CI 4.18-6.70, p < 0.001). Contrastingly, knowledge was significantly lower among divorced (OR = 0.55, 95%CI 0.31-0.96, p = 0.036) and unemployed caregivers (OR = 0.54, 95%CI 0.37-0.80, p = 0.002), as well as participants residing in the northern region (OR = 0.65, 95%CI 0.46-0.91, p = 0.013) and those who had an aunt/uncle diagnosed with diabetes (OR = 0.57, 95%CI 0.40-0.83, p = 0.003) (Table 4)

**Table 3:** Factors associated with caregivers’ knowledge about diabetic foot. All values are presented as numbers (N) and percentages (%); P value<0.05 is statistically significant

Parameter	Category	Knowledge about diabetic foot				p
		No= 673		Yes, =1,248		
		N	%	N	%	
Age (years)	18 to <30	270	35.2%	497	64.8%	0.254
	30 to <45	238	37.2%	401	62.8%	
	45 to <60	148	32.7%	305	67.3%	
	60 or more	17	27.4%	45	72.6%	
Gender	Male	283	38.3%	455	61.7%	0.016
	Female	390	33.0%	793	67.0%	
Nationality	Saudi	631	34.8%	1,180	65.2%	0.476
	Non-Saudi	42	38.2%	68	61.8%	
Marital status	Single	235	35.6%	426	64.4%	<0.001
	Married	369	32.8%	757	67.2%	
	Divorced	46	52.9%	41	47.1%	
	Widow	23	48.9%	24	51.1%	
Region	Eastern	145	33.6%	287	66.4%	0.003
	Western	71	26.5%	197	73.5%	
	Northern	131	39.0%	205	61.0%	
	Southern	109	33.0%	221	67.0%	
	Central	217	39.1%	338	60.9%	
Employment status	Student	138	31.9%	295	68.1%	<0.001
	Unemployed	210	42.0%	290	58.0%	
	Employed	325	32.9%	663	67.1%	
Educational level	Primary	21	42.0%	29	58.0%	<0.001
	Middle	36	56.2%	28	43.8%	
	Secondary	152	38.3%	245	61.7%	
	Diploma	94	40.9%	136	59.1%	
	Bachelor	346	34.4%	661	65.6%	
	Post-graduate	24	13.9%	149	86.1%	
Relationship	Parent	248	32.2%	523	67.8%	0.025
	Sibling	60	35.9%	107	64.1%	
	Child	100	35.0%	186	65.0%	
	Aunt or uncle	89	44.5%	111	55.5%	
	Grandmother/ Grandfather	97	36.6%	168	63.4%	
	Others	79	34.0%	153	65.9%	
Was the patient diagnosed with diabetic foot?	No	540	43.0%	717	57.0%	<0.001
	Yes	133	20.0%	531	80.0%	
What is the condition of the patient feet at the current time	Healthy	580	36.6%	1,004	63.4%	<0.001
	Bad	59	36.2%	104	63.8%	
	Amputated	34	19.5%	140	80.5%	
Is the patient a smoker	No	544	35.6%	985	64.4%	0.323
	Yes	129	32.9%	263	67.1%	
Do you have any experience in dealing with diabetic foot?	No	527	53.5%	458	46.5%	<0.001
	Yes	146	15.6%	790	84.4%	

**Table 4:** Results of the independently associated factors with caregivers’ knowledge about diabetic foot based on the regression analysis. P value<0.05 is statistically significant

Parameter	Category	OR	95% CI	p
Gender	Male	—	—	
	Female	1.42	1.12, 1.81	0.004
Marital status	Single	—	—	
	Married	1.32	0.98, 1.79	0.071
	Divorced	0.55	0.31, 0.96	0.036
	Widow	0.66	0.32, 1.37	0.263
Region	Eastern	—	—	
	Western	1.26	0.87, 1.85	0.225
	Northern	0.65	0.46, 0.91	0.013
	Southern	0.84	0.60, 1.19	0.338
	Central	0.78	0.58, 1.06	0.112
Employment status	Student	—	—	
	Unemployed	0.54	0.37, 0.80	0.002
	Employed	0.73	0.51, 1.04	0.080
Educational level	Primary	—	—	
	Middle	0.52	0.21, 1.23	0.138
	Secondary	1.53	0.75, 3.09	0.234
	Diploma	1.28	0.61, 2.66	0.506
	Bachelor	1.69	0.84, 3.34	0.137
	Post-graduate	4.92	2.15, 11.3	<0.001
Relationship	Parent	—	—	
	Sibling	0.77	0.52, 1.16	0.212
	Child	0.96	0.69, 1.33	0.793
	Aunt or uncle	0.57	0.40, 0.83	0.003
	Grandmother / Grandfather	0.82	0.58, 1.16	0.258
	Others	0.85	0.58, 1.26	0.420
Do you have a personal history of diabetes	No	—	—	
	Yes	2.18	1,05, 4,82	0,044
Was the patient diagnosed with diabetic foot?	No	—	—	
	Yes	2.37	1.78, 3.17	<0.001
What is the condition of the patient feet at the current time	Healthy	—	—	
	Bad	0.66	0.43, 1.01	0.055
	Amputated	1.44	0.91, 2.31	0.129
Do you have any experience in dealing with diabetic foot?	No	—	—	
	Yes	5.28	4.18, 6.70	<0.001

## Discussion

Diabetic foot syndrome is a multifaceted clinical condition that is often accompanied by neuropathy, osteomyelitis, DFU, and ultimately, amputation [3]. This syndrome imposes a significant burden on patients, caregivers, and the healthcare industry [4-6]. In Saudi Arabia, the incidence rate of diabetic foot syndrome is approximately 1.8%, with a mean frequency of 8.5% [10].

Various preventive measures have been shown to reduce the incidence of diabetic feet and the need for LLA through collaborative efforts between patients and caregivers [12]. Therefore, the objective of our study was to evaluate the knowledge and skills of caregivers in Saudi Arabia who provide care to patients with diabetic foot syndrome.

We found that the demographic data of the caregivers indicated that approximately 39.9% had a mean age of 35.5 ± 12.4, which is consistent with a study conducted in Egypt measuring the knowledge of caregivers on how to deal with diabetic foot patients undergoing amputation. They reported that the mean age of caregivers was 37.21±13.87 [14]. This finding is also in line with a study conducted in Northern Portugal in 2018 measuring the hazards of life among caregivers of diabetic patients who underwent amputation, where they reported that the average age of caregivers was 51.74±15.16 [6]. This indicates that younger populations have a higher ability to provide care for their relatives. Regarding the gender of caregivers for patients with diabetic feet, the majority were females, which is consistent with the study conducted in Egypt by Abdel-Mordy et al., where most of the caregivers were females too [14]. The study also revealed that the majority of caregivers were sons and daughters who provided care to their parents, which contradicts the results of the previous study conducted in Egypt, where most caregivers were wives who provided care to their husbands [14]. Both these findings support the theory that caregivers tend to reside with their patients in the same household.

Regarding the marital status of caregivers in our study, we found that the majority were married, which is consistent with the findings of Abdel-Mordy et al. [14] and Wang et al. [11]. In terms of occupation, more than half of the participants in our study were employed, which differs from the findings of Abdel-Mordy et al. [14] as their study mostly consisted of housewives. Furthermore, with regard to the educational level, our study found that most caregivers were graduates with bachelor's degrees, which contradicts the findings of Abdel-Mordy et al. [14], who found that most of their sample had only a secondary school education.

The present study found that the majority of participants were accustomed to biweekly foot checks, consistent cleaning, moisturizing, and drying of the feet, regular nail trimming, preventing the patient from walking barefoot, and ensuring they wear shoes with socks. The most commonly worn footwear was medical shoes with cotton socks. These findings differ from those of El-Rahman et al., who reported that prior to receiving an educational program, most of their sample population did not know the importance of regular foot checks, patients' feet were not kept clean or moisturized, and nail trimming was done solely by the patient [16]. However, after the implementation of the program, substantial improvement in care levels was observed, with caregivers becoming more involved in patient care. These results underscore the significance of implementing educational programs for diabetic patients' caregivers regarding proper foot care routines. In terms of total knowledge and experience, further investigation is required.

Our study found a statistically significant relationship between female gender, marital status, personal history of diabetes, and higher levels of knowledge and experience in dealing with DFUs among caregivers (Table 4). This is consistent with the findings of previous studies, which have demonstrated that female caregivers are more likely to be involved in caring for diabetic patients, and that personal experience with diabetes can lead to a greater understanding of the condition [14,17]. However, our results contradict those of Kenchetty and George, who reported a high prevalence of poor knowledge among caregivers in their study [17]. Our study's findings suggest that providing education and training to caregivers can improve their knowledge and experience in dealing with DFUs, thereby improving patient outcomes [16]. Overall, the caregivers in our study demonstrated a fair level of knowledge about DFUs (Table 3).

According to our study, the majority of caregivers providing care to patients with diabetic foot in Saudi Arabia were female, married, employed, and had a bachelor's degree. Furthermore, they exhibited adequate knowledge and experience in managing DFUs. To enhance foot care among patients with diabetic foot, we recommend implementing a comprehensive health education program that employs multiple strategies, such as lectures and informative materials, to improve patients' understanding and adherence to proper foot care practices. Additionally, we suggest utilizing mass media channels to disseminate information on the importance of regular self-foot inspection to a wider audience. Moreover, we recommend implementing a program that aims to enhance caregivers' knowledge and skills in caring for diabetic foot patients, which should be available within medical facilities catering to diabetes patients.

However, it is essential to acknowledge that our study had several limitations. Firstly, our participants were drawn from a relatively narrow socioeconomic group, which limits the generalizability of our findings to their respective families. Secondly, the study was relatively short in duration making it difficult to generalize the results to all communities.

## Conclusions

According to our investigation, it appears that individuals who provide care for patients with diabetic foot in Saudi Arabia are predominantly young, female, married, and educated. A substantial proportion of diabetic patients experience diabetic foot, with a small subset demonstrating poor status or requiring amputation. Caregivers routinely conduct regular foot examinations, maintain cleanliness and moisturization, trim nails, and ensure that patients wear suitable footwear. Many caregivers possess experience managing diabetic foot patients and are knowledgeable about foot complications in diabetic patients. Factors associated with increased knowledge include being female, holding a postgraduate degree, having a personal history of diabetes, providing care for patients with diabetic foot, and having prior experience with diabetic foot patients. Conversely, factors associated with decreased knowledge include being divorced, unemployed, and residing in the northern region of the country. Overall, these results suggest that caregivers of diabetic foot patients in Saudi Arabia exhibit good knowledge and practice in relation to foot care. However, disparities may exist in knowledge and experience among various subsets of caregivers.
